# Targeting estrogen receptor beta (ERβ) for treatment of ovarian cancer: importance of KDM6B and SIRT1 for ERβ expression and functionality

**DOI:** 10.1038/s41389-018-0027-9

**Published:** 2018-02-09

**Authors:** Giulia Pinton, Stefan Nilsson, Laura Moro

**Affiliations:** 1 Department of Pharmaceutical Sciences, University of Piemonte Orientale “A. Avogadro”, 28100 Novara, Italy; 2 Department of Biosciences and Nutrition, Karolinska Institutet, S-141 57 Huddinge, Sweden

## Abstract

Estrogen receptor (ER) β has growth inhibitory and chemo drug potentiating effect on ovarian cancer cells. We studied the dependence of ERβ function on the presence of KDM6B and SIRT1 in human ovarian cancer cells in vitro. Activation of ERβ with the subtype-selective agonist KB9520 resulted in significant inhibition of human ovarian cancer cell growth. KB9520-activated ERβ had an additive effect on growth inhibition in combination with cisplatin and paclitaxel, respectively. Loss of KDM6B expression had a negative effect on ERβ function as a ligand-dependent inhibitor of ovarian cancer cell growth. In contrast, loss or inhibition of SIRT1 deacetylase activity restored ligand-activated ERβ functionality. Presented data suggest that selective targeting of ERβ with an agonist potentiate chemotherapy efficacy for the treatment of ovarian cancer and that downregulation or inhibition of SIRT1 may further enhance its therapeutic effect.

## Introduction

Ovarian epithelial carcinoma (OEC), believed to originate from the ovarian surface epithelium, is the fourth commonest cause of female cancer death in the developed world. The OEC incidence is estimated to more than 240,000 new cases and around 150,000 deaths per year worldwide. The highest rates are reported in Scandinavia, Eastern Europe, USA, and Canada^1–4^.

The etiology of OEC is poorly understood but it is believed that nulliparity, high plasma levels of estrogen or long-term estrogen replacement therapy increases the risk for ovarian cancer, whereas pregnancy, lactation, and oral contraceptives decrease the risk^1, 4–7^.

The standard of care for the management of advanced ovarian cancer has been unchanged for many years and includes maximum cytoreductive surgery followed by platinum-based chemotherapy (carboplatin or cisplatin) in combination with paclitaxel^2, 8^. Although the response rate for first-line carboplatin and paclitaxel is high, approximately 80% of patients with advanced OEC will experience recurrence and eventually become resistant to chemotherapy^2,8, 9^. New investigational drugs targeting different pathways for improved disease control and for the treatment of platinum-resistant OEC are emerging^10^.

Both estrogen receptor (ER) subtypes, ERα and ERβ, are expressed in normal ovarian tissue as well as in ovarian cancer cells^7, 11^. There is, however, a falling trend in ERβ expression as cells transition from normal to malignant state and with a further decline in its expression as the malignancy develops from early (stage I) to late disease stages (stage II–IV). No such trend in the expression of ERα has been observed^7, 11^.

The levels of ERα are closely associated with estrogen-dependent growth and to increased metastatic potential of OEC by promoting epithelial–mesenchymal transition (EMT) through upregulation of Snail and Slug and downregulation of E-cadherin. In contrast, ERβ mediates opposite effects to ERα in the presence of 17β-Estradiol (E2), resulting in inhibition of EMT^12^.

Although 40–60% of ovarian cancers express ERα, only a portion (15–18%) of them benefit from anti-estrogen (SERM) treatment, if at all. Tamoxifen produces only a modest effect in ovarian cancer^1,11, 13^.

Introduction of ERβ into the ERα-positive human epithelial ovarian cancer cell line BG-1 led to decreased basal and E2-induced cell proliferation in vitro. Moreover, ERβ expression resulted in downregulation and inhibition of ERα activity, arrested the cells in the G2/M phase, and decreased the pAKT levels^13^.

Treatment of the ovarian cancer cell lines SKOV3 and OV2008 with the ERα-selective agonist, PPT, led to a significant stimulation of cell growth. In contrast, the ERβ-selective agonist, DPN, significantly suppressed the growth of the two ovarian cancer cells. Moreover, the size of tumors in the in vivo SKOV3 xenograft model were significantly smaller in DPN-treated compared to vehicle-treated animals^14^. A recent study provided evidence that also natural ERβ agonists have the potential to reduce cell viability and survival and to promote apoptosis of OEC cells^15^.

ERβ expression in ovarian cancer cells has been found to be significantly associated with longer disease-free survival and overall survival (OS)^11^. Moreover, patients with more than 30% of ERβ-positive cancer cells were shown to respond well to chemotherapy and with increased progression-free survival and OS compared to patients with less than 30% of ERβ-positive tumor cells^1^. The analysis also demonstrated significantly longer OS time of patients with higher ERβ immunoreactivity score after chemotherapy, compared with patients with low ERβ score.

The recent discovery of alterations in genes encoding histone modifiers suggests their possible roles in cancer development. The lysine-specific demethylase 6B (KDM6B), also called Jumonji domain-containing protein D3 (Jmjd3), is a member of the family of JmjC histone demethylases that specifically catalyzes the demethylation of di-methylated or tri-methylated Lys 27 in histone H3 (H3K27me2/3). The role of KDM6B has been extensively studied in development, cell plasticity, immune system, neurodegenerative disease, and cancer^16^.

While, H3K27me3 is considered a repressive epigenetic mark and is recognized as a determining factor in promoting tumorigenesis and tumor progression, the mechanisms underlying KDM6B expression and function in cancer is still controversial^17^.

Accumulating evidence indicates that SIRT1, involved in a variety of cellular processes, is a key player in oncogenesis and cancer progression. SIRT1 belongs to a family of seven class III histone deacetylases (HDACs) enzymes, which are highly conserved enzyme homologs of the yeast Sir2 protein, with nicotine adenine dinucleotide NAD+-dependent protein deacetylase activity^18^. Overexpression of SIRT1 has been recently associated with poor outcome and chemoresistance of OEC patients^19–22^.

In this report we have studied the role and effect of ERα and ERβ in response to the natural hormone estradiol (E2) and the subtype-selective agonists PPT (ERα-selective) and KB9520 (ERβ-selective) in the human ovarian cancer cell lines SKOV3 and A2780cis. Furthermore, we have characterized the role of KDM6B and SIRT1 for the tumor inhibitory mechanism of ERβ in the presence of its selective agonist KB9520.

Altogether, the preclinical and clinical data on OEC suggest that selective targeting of ERβ may provide a significant improvement of existing therapy for the treatment of ovarian cancer, at least for the patient population that expresses ERβ.

## Results

### ER subtype expression and response to ligands

**Fig. 1:**
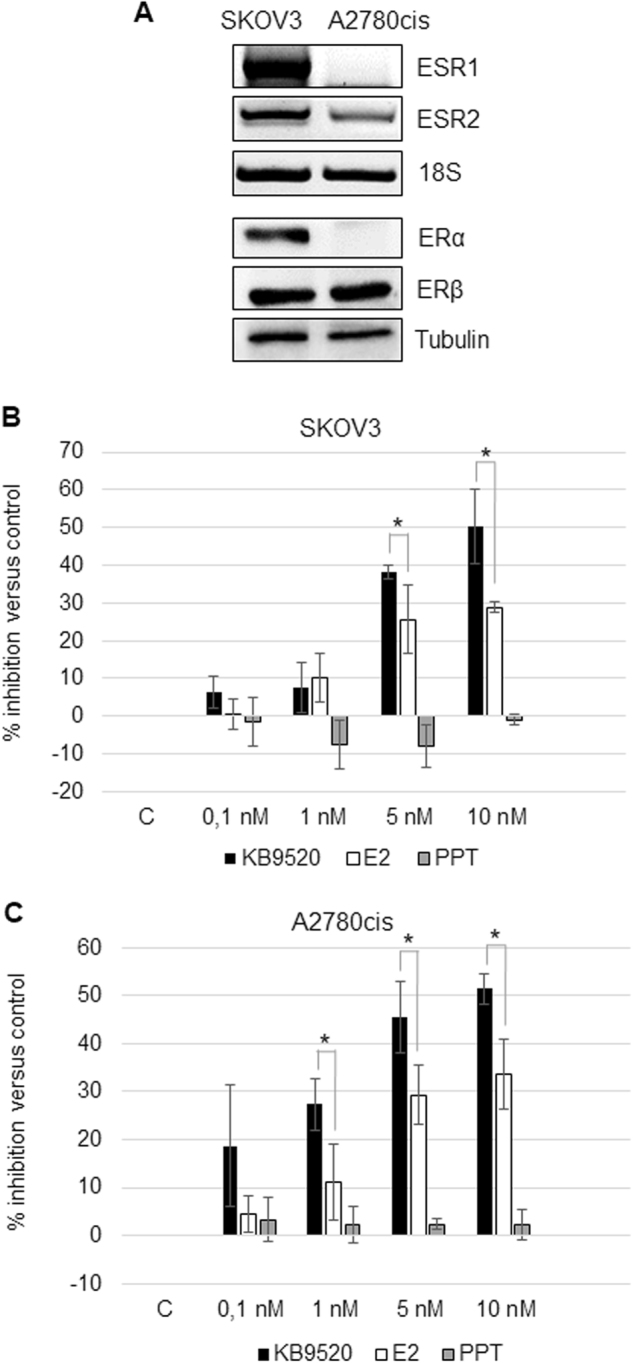
Estrogen receptor subtype expression and response to ligands. **a** Representative RT-PCR and Western blot analyses of *ESR1* and *ESR2* expression in SKOV3 and A2780cis cells. 18S rRNA and Tubulin were used as controls. **b** Percentage of growth inhibition in SKOV3 and **c** A2780cis cells after 24-h treatment with different doses of KB9520, E2, or PPT, in the range of 0.1 to 10 nM. Results are expressed as mean ± s.d. of three independent experiments. *p ≤ 0.05

**Fig. 2:**
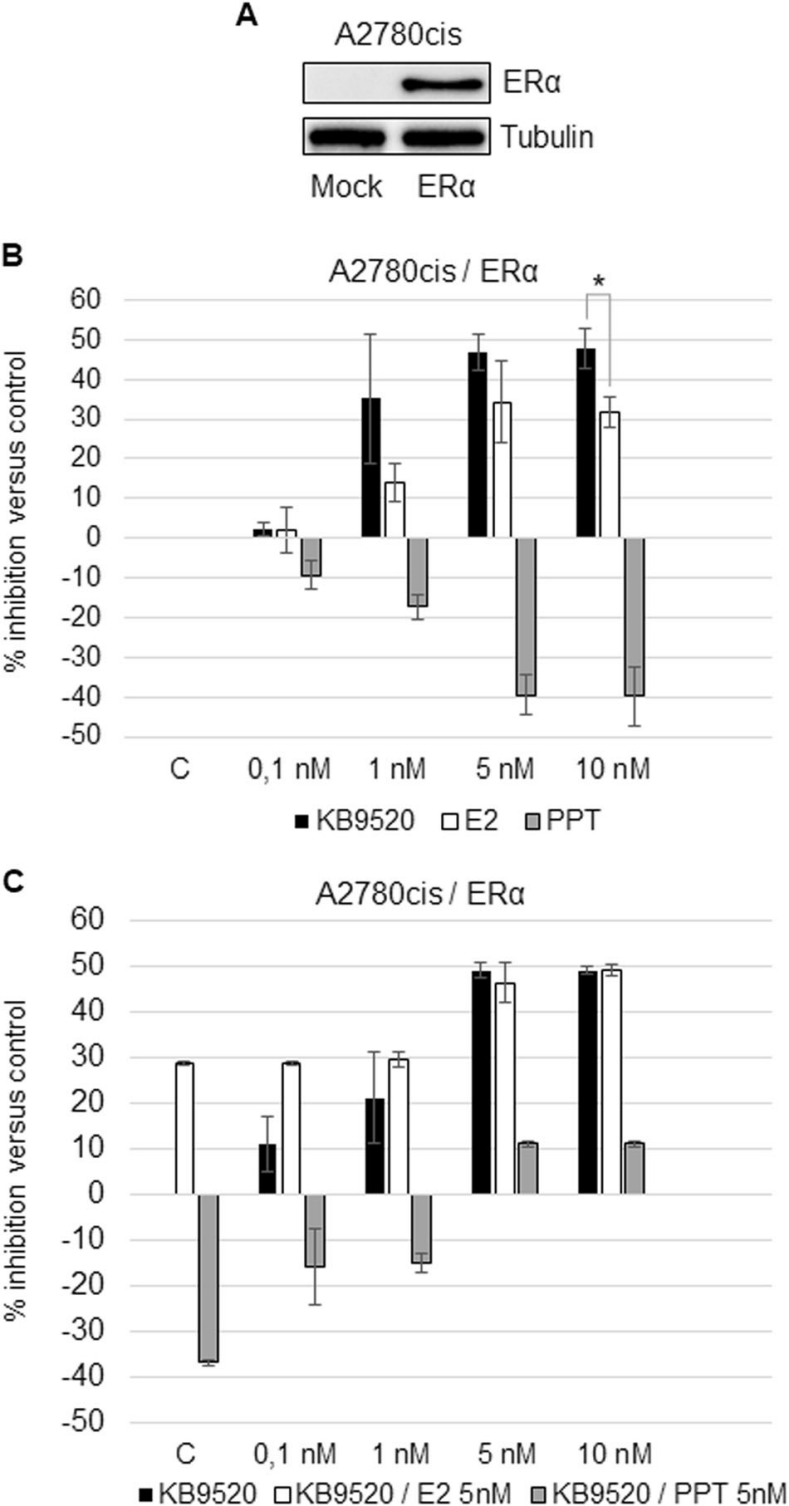
Response to ligands of ERα-transfected A2780cis cells. **a** Representative Western blot analysis of ERα expression in Mock and pcDNA3.1-ERα-transfected A2780cis cells. Tubulin was used as loading controls. **b** Percentage of growth inhibition in ERα-transfected A2780cis cells after 24-h treatment with different doses of KB9520, E2, or PPT, in the range of 0.1 to 10 nM. **c** Percentage of growth inhibition in ERα-transfected A2780cis cells after 24-h treatment with different doses of KB9520, in the range of 0.1 to 10 nM, in combination with 5 nM E2 or PPT. Results are expressed as mean ± s.d. of three independent experiments. *p ≤ 0.05

The expression of the two ER subtypes, ERα and ERβ, was investigated in the human ovarian cancer cell lines SKOV3 and A2780cis, respectively. The SKOV3 cells were shown to express both ER subtypes at gene and protein levels, whereas the A2780cis cells only expressed ERβ (Fig. 1a). The effect of the non-subtype-selective agonist E2 and the two ER subtype-selective ligands KB9520 and PPT on cell growth was explored (Fig. 1b, c). The ERβ-selective agonist KB9520 resulted in a robust, concentration-dependent, inhibition of cell growth of both SKOV3 and A2780cis cells, with a maximum at 5–10 nM. The ERα-selective agonist PPT caused only a weak concentration-dependent stimulation of SKOV3 cell growth, whereas the growth of the ERα-negative A2780cis cells was unaffected. E2 inhibited the growth of A2780cis cells in a concentration-dependent fashion, and unexpectedly, of also SKOV3 cells, with a similar efficacy as the ERβ-selective agonist KB9520. This efficacious and unexpected cell growth inhibitory effect of E2 in the SKOV3 cells may, however, be explained by the reported loss-of-function mutation of ERα in these cells^23^. Because of the mutated ERα in SKOV3 cells, we transfected A2780cis cells with a wild-type ERα expression vector (Fig. 2a). As shown in Fig. 2b, PPT resulted in a robust stimulation of A2780cis/ERα cell growth as compared to SKOV3 cells (Fig. 1b), with maximum stimulation at a concentration of 5–10 nM. E2 still caused inhibition of cell growth, similar to that of KB9520, despite the expression of wild-type ERα (Fig. 2b). In combination with a fixed concentration of PPT (5 nM), KB9520 inhibited the growth stimulatory effect of PPT in a concentration-dependent fashion (Fig. 2c). Combination of a fixed concentration of E2 with increasing concentrations of KB9520 resulted in an additive effect on cell growth inhibition.

### Agonist-activated ERβ destabilizes the ERα protein

**Fig. 3:**
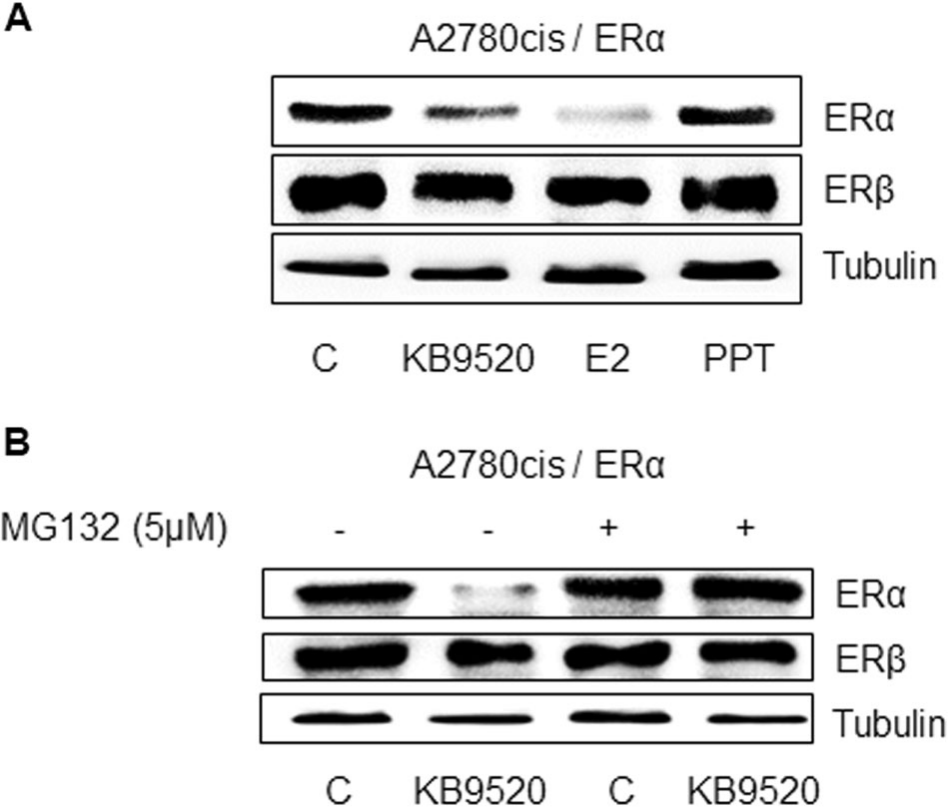
Agonist-activated ERβ destabilizes the ERα protein. **a** Representative Western blot analyses of ERα and ERβ expression in ERα-transfected A2780cis cells after 24-h treatment with 10 nM of KB9520, E2, or PPT. **b** Representative Western blot analyses of ERα and ERβ expression in ERα-transfected A2780cis cells after 24-h treatment with 10 nM of KB9520 alone or in combination with 5 nM MG132. Tubulin was used as loading control

It has previously been reported that ERβ has an inhibitory effect on ERα expression and activity in BG-1 epithelial ovarian cancer cells^12, 13^. To explore the potential mechanism of the inhibitory effect of ERβ on ERα activity in A2780cis cells, we treated the A2780cis/ERα cells with E2 and KB9520 for 24 h. As shown in Fig. 3a, the ERβ-selective agonist KB9520 and the non-ER subtype-selective agonist E2 resulted in decreased ERα protein levels as compared to PPT and untreated A2780cis/ERα cells. To further investigate this effect of ERβ on ERα protein stability, the A2780cis/ERα cells were treated with KB9520 in the absence or presence of the proteasome inhibitor MG132 (Fig. 3b). Treatment with MG132 resulted in stabilization of the ERα protein at control levels in the presence of KB9520, suggesting that ERβ plays a role in proteasome-mediated ERα protein degradation.

### Additive effect of KB9520 on cisplatin and paclitaxel sensitivity in A2780cis and SKOV3 cells

**Fig. 4:**
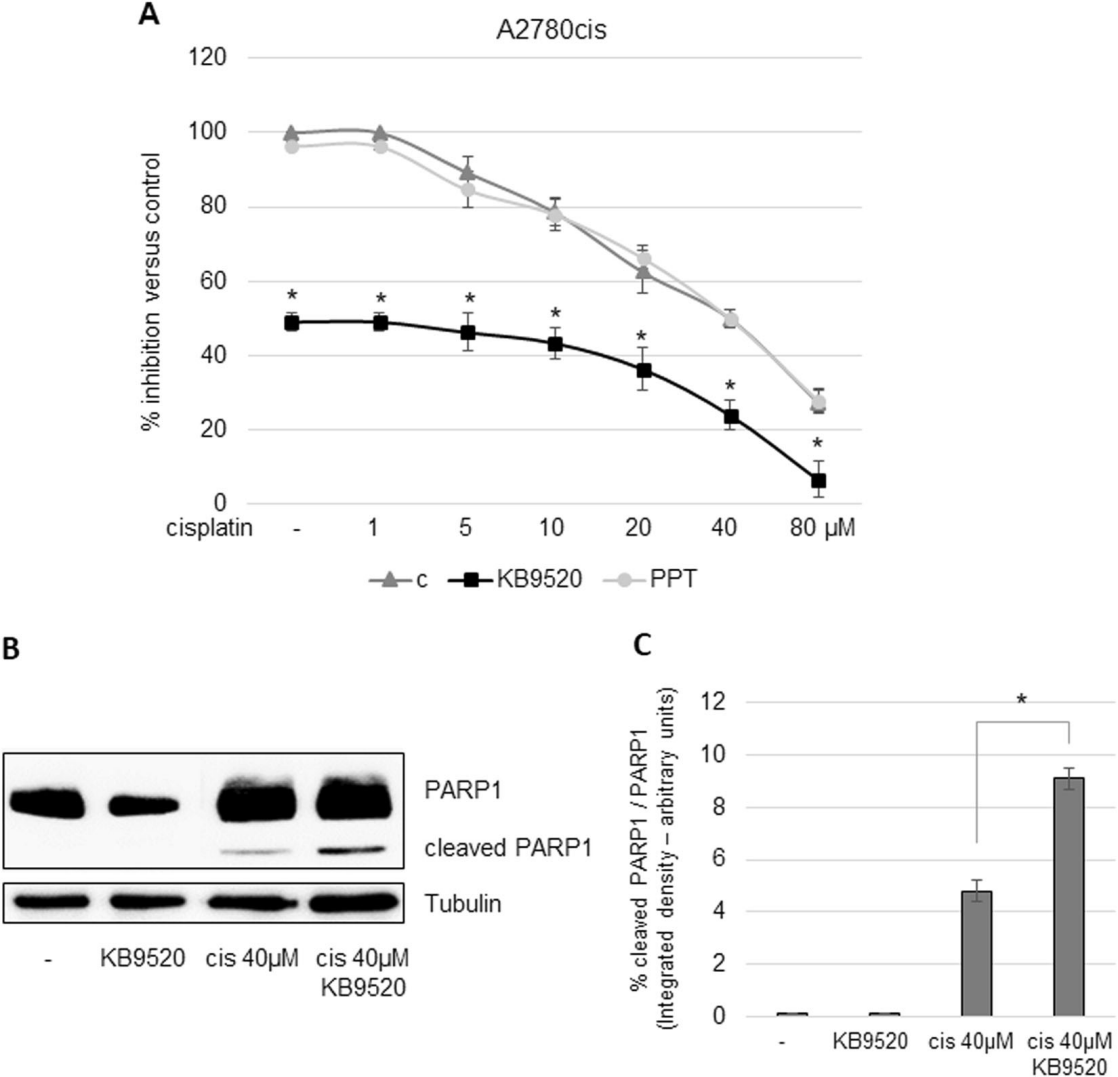
Additive effect of KB9520 on cisplatin sensitivity in A2780cis cells. **a** Percentage of growth inhibition in A2780cis cells after 24-h treatment with different doses of cisplatin, in the range of 1 to 80 μM alone or in combination with 10 nM of KB9520 or PPT. Results are expressed as mean ± s.d. of three independent experiments. *p ≤ 0.05. **b** Representative Western blot and **c** densitometric analyses of PARP1 cleavage in A2780cis cells after 24-h treatment with 40 μM of cisplatin alone or in combination with 10 nM of KB9520. *p ≤ 0.05. Tubulin was used as loading control

The ERβ-selective agonist KB9520 had a synergistic effect on cisplatin sensitivity in the malignant pleural mesothelioma REN cell line in vitro and in vivo^24^. In this study, treatment of A2780cis and SKOV3 cells with a fixed concentration of KB9520, on top of increasing concentrations of cisplatin, resulted in an additive effect over the full range of cisplatin concentrations (Fig. 4a, S1A, B), whereas PPT was without effect. Treatment with E2 also resulted in an additive effect in combination with cisplatin but less pronounced than KB9520 (data not shown). KB9520 treatment also increased the level of cleaved PARP1 in combination with cisplatin (Fig. 4b, c).

Additive growth inhibition of A2780cis cells was also observed when cells were treated with KB9520 in combination with paclitaxel (Fig. S2).

### Role of KDM6B and SIRT1 for the expression and function of ERβ and its response to KB9520 in A2780cis cells

**Fig. 5:**
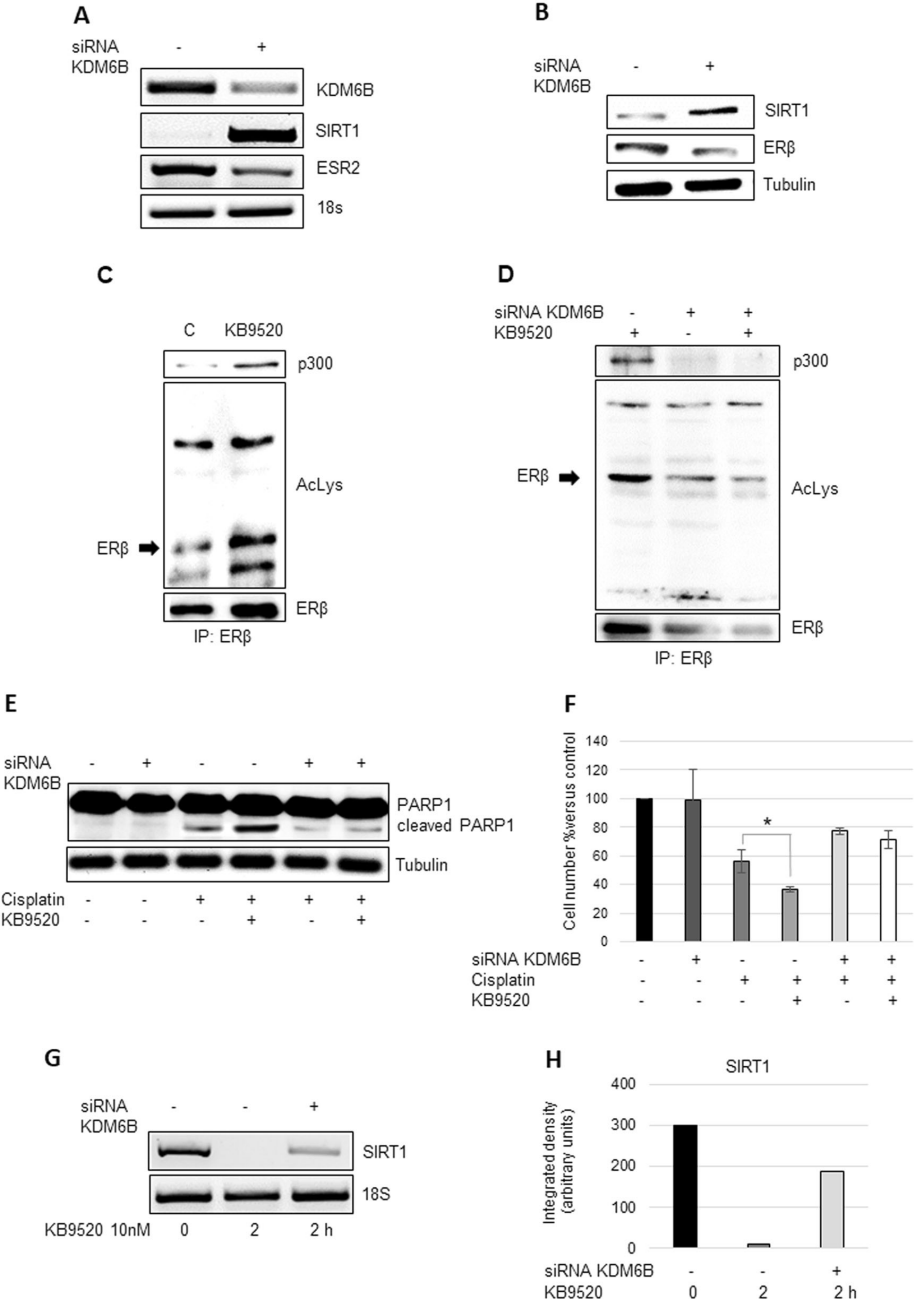
Role of KDM6B for the expression and function of ERβ and its response to KB9520 in A2780cis cells. **a** Representative RT-PCR and **b** Western blot analyses of *KDM6B*, *SIRT1*, and *ESR2* expression in A2780cis cells transfected 48 h with non-specific or *KDM6B*-specific siRNAs. 18S rRNA and Tubulin were used as controls. **c** Immunoprecipitation of ERβ, from lysates of A2780cis cells treated or not with 10 nM of KB9520, 2 h; lysine acetylation and co-immunoprecipitated proteins were detected by Western blot analyses using the respective antibodies (AcLys, ERβ, and p300). **d** Immunoprecipitation of ERβ, from lysates of A2780cis cells transfected 48 h with non-specific or *KDM6B*-specific siRNAs and treated or not with 10 nM of KB9520, 2 h; lysine acetylation and co-immunoprecipitated proteins were detected by Western blot analyses using the respective antibodies (AcLys, ERβ, and p300). **e** Representative Western blot analysis of PARP1 cleavage in A2780cis cells transfected with non-specific or *KDM6B-*specific siRNAs after 24 h treatment with 40 μM of cisplatin alone or in combination with 10 nM of KB9520. Tubulin was used as loading control. **f** Percentage of viable A2780cis cells transfected with non-specific or *KDM6B*-specific siRNAs after 24 h treatment with 40 μM of cisplatin alone or in combination with 10 nM of KB9520. Results are expressed as mean ± s.d. of three independent experiments. *p ≤ 0.05. **g** Representative RT-PCR, and **h** respective densitometry, of *SIRT1* expression in A2780cis cells transfected with non-specific or *KDM6B*-specific siRNAs for 48 h and then treated or not with 10 nM of KB9520 for 2 h. 18S rRNA was used as housekeeping gene

We have previously reported an important role for the Jumonji domain containing 3 histone demethylase KDM6B for the expression of ERβ in human epithelioid and biphasic pleural mesothelioma cell lines^25^. In this report we observed that KB9520-activated ERβ increased the levels of *KDM6B* almost 20-fold, whereas the expression of the methyltransferase *EZH2* was unaffected (Fig. S3). Knock-down of *KDM6B* affected the function of ERβ as a ligand-dependent transcription factor. siRNA inhibited expression of *KDM6B* resulted in increased expression of SIRT1, at both the mRNA and protein levels, and decreased transcripts of the ERβ gene (*ESR2*), with a subsequent consequence on the ERβ protein level (Fig. 5a, b). Whereas acetylation of ERβ and complex formation with p300 was increased in a KB9520-dependent fashion in control cells (Fig. 5c), knock-down of KBM6B led to decreased acetylation of the ERβ protein and its complex formation with p300, irrespective of the presence or absence of KB9520 (Fig. 5d). In addition, the cellular sensitivity to cisplatin and the additive effect of KB9520 was lost (Fig. 5e, f, S4). Also the KB9520-mediated inhibition of *SIRT1* expression was significantly reduced following knock-down of *KDM6B* (Fig. 5g, h). KB9520 did not induce ERα acetylation and, in addition, led to reduced p300:ERα association (Fig. S5).

**Fig. 6:**
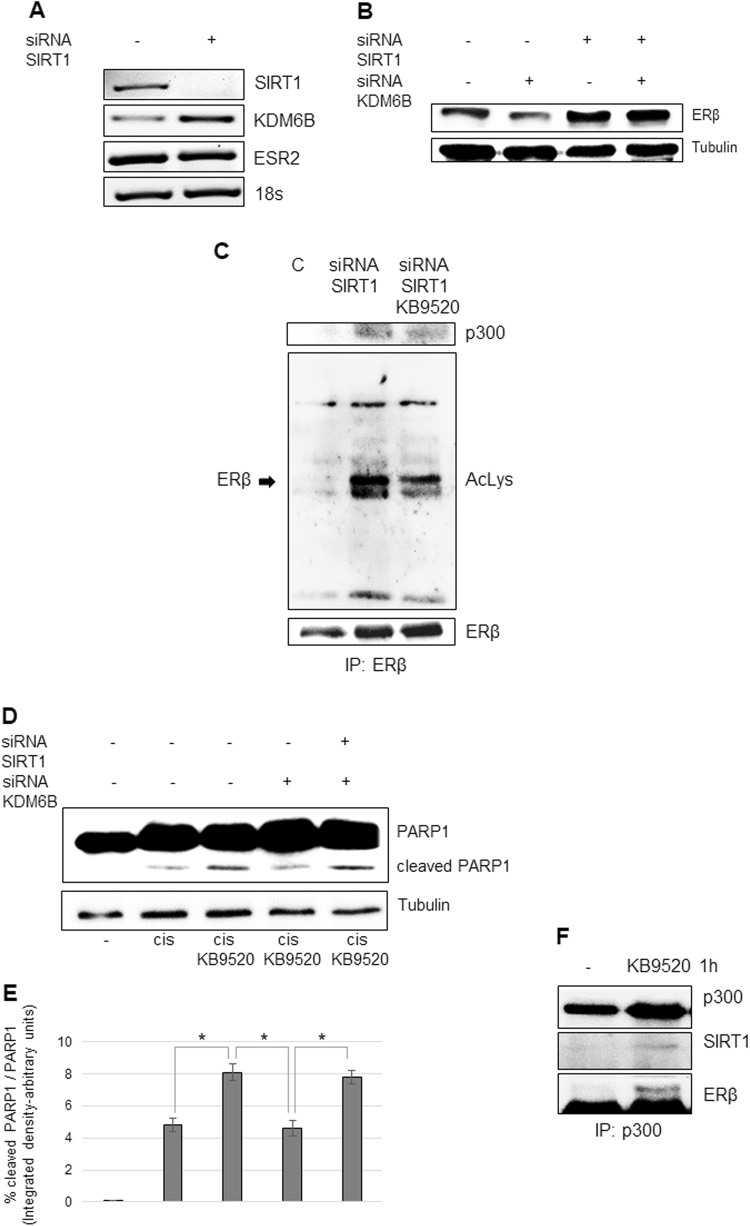
Role of SIRT1 for the expression and function of ERβ and its response to KB9520 in A2780cis cells. **a** Representative RT-PCR of *SIRT1*, *KDM6B*, and *ESR2* expression and **b** Western blot analyses of ERβ expression in A2780cis cells transfected with non-specific or *SIRT1 and KDM6B*-specific siRNAs. 18S rRNA and Tubulin were used as controls. **c** Immunoprecipitation of ERβ, from lysates of A2780cis cells transfected for 48 h with non-specific or *SIRT1*-specific siRNAs and then treated or not with 10 nM of KB9520, 2 h; lysine acetylation and co-immunoprecipitated proteins were detected by Western blot analyses using the respective antibodies (AcLys, ERβ, and p300). **d** Representative Western blot and **e** densitometric analysis of PARP1 cleavage in A2780cis cells transfected with non-specific or *SIRT1* and *KDM6B*-specific siRNAs after 24 h treatment with 40 μM of cisplatin alone or in combination with 10 nM of KB9520. Tubulin was used as loading control. *p ≤ 0.05. **f** Immunoprecipitation of p300, from lysates of A2780cis treated or not 1 h with 10 nM of KB9520; co-immunoprecipitated proteins were detected by Western blot analyses using the respective antibodies (p300, SIRT1, and ERβ)

Inhibition of *SIRT1* expression led, on the other hand, to increased *KDM6B* expression and increased levels of ERβ protein, without increased transcription of the *ESR2* gene (Fig. 6a, b). Absence of SIRT1 expression and activity (by gene silencing or inhibition of protein catalytic activity by EX527) resulted in increased ligand-dependent acetylation of ERβ and re-association with acetylated p300 (Fig. 6c, S6A, B). Moreover, SIRT1 depletion restored sensitivity to KB9520 in combination with cisplatin (Fig. 6d, e). To strengthen our suspicion that SIRT1 has a more direct role in the deacetylation of ERβ, A2780cis cells were treated with and without KB9520 for a shorter time point than 2 h. Cell extracts immunoprecipitated with a p300 antibody showed that SIRT1 exists in a complex with p300 and ERβ and that this association is ligand dependent (Fig. 6f).

## Discussion

The standard of care for the management of advanced ovarian cancer has been unchanged for many years and includes maximum cytoreductive surgery followed by platinum-based chemotherapy (carboplatin or cisplatin) in combination with paclitaxel^2, 8^. Although the response rate for first-line carboplatin and paclitaxel is 70–80%, the majority of women with advanced ovarian cancer will relapse or progress and eventually develop chemotherapy-resistant disease^2, 9^. Clinical trials in the search for innovative, targeted therapies for treatment of ovarian cancer is ongoing but so far no standard second-line treatment stands out as superior with regard to efficacy or safety.

It has previously been reported that ERβ mediates ovarian cancer cell growth repression by decreasing the cellular content of, among others, retinoblastoma, phospho-AKT, cyclin D1, and A2 as well as upregulating the cyclin-dependent kinase inhibitor p21^13,14,26, 27^. In this report we have focused on other aspects of ERβ function and activity as a ligand-activated transcription factor and growth inhibitor of tumor cells.

We initiated the studies on the human ovarian cancer cell lines, SKOV3 and A2780cis, by determining their expression of ERα and ERβ, respectively, and by exploring the effect of the non-subtype-selective, natural hormone E2, which binds to ERα and ERβ with similar affinity, and the two subtype-selective agonists KB9520 (ERβ-selective) and PPT (ERα-selective), on the growth of these cells (Fig. 1). In the presence of both ER subtypes, we could show that KB9520 had a dominant inhibitory effect on cell growth, antagonizing the growth stimulatory effect of PPT (Fig. 2), which is in agreement with published results on DPN and PPT on the mouse mammary cell line HC11^28^. Perhaps less expected was that E2 also exerted a dominant cell growth inhibition, with the same efficacy as KB9520, despite the presence of also ERα (Fig. 2). This result is in disagreement with published results on the mouse mammary cell line HC11^28^. This discrepancy may, however, be due to the difference in origin of the cell lines since our E2 results are in agreement with published data in ERβ overexpressed, human ovarian, and breast cancer cell lines that express endogenous ERα; ERβ has a dominant, negative influence on ERα activity^12,13,29, 30^.

That the proteasome inhibitor MG132 prevented loss of the ERα protein, following activation of ERβ with KB9520, suggests that ERβ is responsible for targeting ERα for proteasome pathway degradation (Fig. 3a, b).

Due to the ERβ-mediated loss of the ERα protein, presented data strongly support our notion that the growth inhibition by E2, in the presence of both ER subtypes, is mediated by ERβ. However, also other mechanisms may explain the dominant growth inhibitory effect of ERβ such as: formation of ERα:ERβ heterodimer in the presence of E2 and that ERβ dictates the activity of the heterodimer, competition with ERα for binding to sites on DNA within target genes or competition for interaction with coregulatory proteins (Fig. S5)^29–31^.

Although we received a similar increase in cleaved PARP1 by KB9520 in combination with cisplatin in the A2780cis cells as in the human malignant pleural REN cell line, we did not observe a synergistic effect of cell growth inhibition (Fig. 4, S1)^24^. The mechanistic difference in response, additive vs. synergism, is unknown to us and needs further and deeper investigation.

Posttranslational acetylation of ERα was recently reported to play an important role for its activity as a ligand-activated transcription factor^32^ and for its stability^33^. In this study we can for the first time present data showing that also acetylation of ERβ is important for its function as a ligand-dependent regulator of cellular events (Figs. 5, 6).

That KDM6B is important for ERβ expression was previously reported by us^25^. In this report, we show that KDM6B is also important for ERβ function. Ligand-dependent acetylation of ERβ and its interaction with p300 was lost following depletion of KDM6B (Fig. 5c compared to 5d). Moreover, the ligand-dependent, additive effect of ERβ on PARP1 cleavage in combination with cisplatin vanished (Fig. 5e, f) and the ability of ERβ to downregulate SIRT1 expression in a ligand-dependent fashion was lost (Fig. 5g, h). Whether the transcriptional regulation of the SIRT1 gene by ligand-activated ERβ is direct or indirect through KDM6B needs further studies; KB9520-activated ERβ upregulated *KDM6B* expression approximately 20-fold (Fig. S3).

SIRT1 was found to exist in a complex with ERβ and p300 at the 1 h time point but not at 2 h or later, implying that deacetylation of ERβ is a sequential event (Fig. 6e compared to 6c and S6). We speculate that loss of ERβ acetylation follows SIRT1-mediated deacetylation of lysine residues 1020/1024 within the cell cycle regulatory domain 1 of p300, which thereafter results in reduced p300-mediated ERβ acetylation^32–35^.

Loss of ERβ acetylation and thereby ligand-dependent ERβ function may be due to decreased DNA-binding activity, inability to interact with coregulators for negative or positive transcriptional gene regulation, changed subcellular location or decreased binding affinity for KB9520^32^.

Knock-down of SIRT1 caused increased levels of ERβ protein without increased transcription of the *ESR2* gene. The mechanism for this effect is unclear but may be a result of a stabilized ERβ protein when acetylated.

Which lysine residues in ERβ that are targets for acetylation need further studies. In conclusion, in this report, we have provided data showing that the function and ligand-dependent activity of ERβ, as an ovarian tumor cell growth inhibitor, is dependent of posttranslational acetylation and that the level of KDM6B and the NAD+-dependent deacetylase SIRT1 play important roles in this process. We have also demonstrated that selective activation of ERβ is additive to cisplatin and paclitaxel in the inhibition of ovarian tumor cell growth and that ERβ has a dominant cell regulatory effect over ERα, even in the presence of E2. Moreover, we have shown that KB9520-activated ERβ has a strong stimulatory effect on KDM6B expression, that most likely add to the ERβ-mediated ovarian tumor inhibitory activity. In summary, presented data suggest that selective targeting of ERβ with an agonist potentiate chemotherapy efficacy for the treatment of ovarian cancer and that downregulation or inhibition of SIRT1 may further enhance its therapeutic effect.

## Materials and methods

### Reagents and antibodies

The polyclonal antibodies specific for ERα (H-184), ERβ (H-150), SIRT1, p300 and the monoclonal antibodies specific for PARP1, acetylated-lysine, and α-tubulin were purchased from Santa Cruz Biotechnology (Santa Cruz, CA, USA). Anti-mouse and anti-rabbit IgG peroxidase or FITC-conjugated antibodies, the proteasome inhibitor MG132, the selective SIRT1 inhibitor EX527 and chemical reagents were from Sigma-Aldrich (St Louis, MO, USA). ECL, nitrocellulose membranes, and protein assay kit were from Bio-Rad (Hercules, CA, USA). TRIzol^®^, culture media, sera, and LipofectAMINE transfection reagent were from Thermo Fisher (Waltham, MA, USA). The ERα-selective agonist PPT and 17β-estradiol were from Tocris Bioscience (Bristol, UK). The ERβ-selective agonist KB9520 was originally provided by Karo Bio AB (Huddinge, SE)^24^. Today this ligand is owned by Oasmia Pharmaceuticals AB (Uppsala, SE).

### Cell cultures and transfection

The ovarian cancer SKOV3 and A2780cis cell lines were purchased from Sigma-Aldrich (St Louis, MO, USA). Cells were grown in standard conditions in RPMI medium supplemented with 10% FBS, 100 μg/ml streptomycin, and 10 μg/ml penicillin at 37 °C in a humidified environment containing 5% CO_2_. Mycoplasma infection was excluded by the use of Mycoplasma PlusTM PCR Primer Set kit from Stratagene (La Jolla, CA, USA). Cells grown to 80% confluence in tissue culture dishes were transiently transfected with the pcDNA3.1-ERα plasmid or with specific siRNAs from Qiagen (Hilden, Germany), using the LipofectAMINE reagent as described by the manufacturer.

### Proliferation assays

Cells were seeded at a density of 10 × 10^4^ cells/well in 6-well plates in RPMI medium supplemented with 10% FBS, 100 μg/ml streptomycin, and 10 μg/ml penicillin and incubated overnight at 37 °C in a humidified environment containing 5% CO_2_ to allow adherence. Following treatment cells were trypsinized and stained with Trypan blue. The number of cells considered viable (unstained cells) was counted in a Bürker haemocytometer within 5 min after staining.

### Cell cycle analysis

For cell cycle/apoptosis analysis, 5 × 10^5^ cells were seeded in tissue culture plates and treated with 10 nM KB9520, 40 µM cisplatin, or the combination of the two drugs for 24 h at 37 °C in a 5% CO_2_ atmosphere. After incubation, detached and suspended cells were harvested in complete RPMI and centrifuged at 500 × *g* for 10 min. Pellets were washed with PBS, fixed in ice-cold 75% ethanol at 4 °C, treated with 100 mg/ml RNAse A for 1 h at 37 °C, stained with 25 μg/ml propidium iodide and finally analyzed by using a flow cytometer FACS (Becton Dickinson, San Jose, CA, USA) and Modfit software (Verity Software House, Topsham, ME, USA).

### Cell lysis, immunoprecipitation, and immunoblot

Cells were extracted with 1% NP-40 lysis buffer (1% NP-40, 150 mM NaCl, 50 mM Tris-HCl pH 8.5 mM EDTA, 10 mM NaF, 10 mM Na_4_P_2_O_7_, 0.4 mM Na_3_VO_4_) with freshly added protease inhibitors (10 μg/ml leupeptin, 4 μg/ml pepstatin, and 0.1 Unit/ml aprotinin). Lysates were centrifuged at 13,000 × *g* for 10 min at 4 °C and the supernatants were collected and assayed for protein concentration with the Bio-Rad protein assay method. For immunoprecipitation experiments, 2 mg of extracted protein for each treatment were incubated with specific antibodies for 1 h at 4 °C and 50 µl protein A-Sepharose beads. Proteins were separated by SDS-PAGE under reducing conditions. Following SDS-PAGE, proteins were transferred to nitrocellulose, reacted with specific antibodies and then detected with peroxidase-conjugate secondary antibodies and chemioluminescent ECL reagent. Digital images were taken with the Bio-Rad ChemiDocTM Touch Imaging System and quantified using Bio-Rad Image Lab 5.2.1.

### RNA isolation and RT-PCR

Total RNA was extracted using TRIzol^®^ reagent. Starting from equal amounts of RNA, cDNA used as template for amplification in the RT-PCR (5 µg), was synthesized by the reverse transcription reaction using RevertAid Minus First Strand cDNA Synthesis Kit from Fermentas-Thermo Scientific (Burlington, ON, Canada), using random hexamers as primers, according to the manufacturer’s instructions. 20 ng of cDNA were used to perform RT-PCR amplification.

The primers sequences were: *ESR1*, Fw 5′-AACAAAGGCATGGAGCATCTGT-3′ and Rev 5′-TGATGTAATACTTTTGCAAGG-3′; *ESR2*, Fw 5′-GTCAGGCATGCGAGTAACAA-3′ and Rev 5′-GGGAGCCCTCTTTGCTTTTA-3′; *KDM6B*, Fw 5′-CCTCGAAATCCCATCACAGT-3′ and Rev 5′-GTGCCTGTCAGATCCCAGTT-3′; *SIRT1*, Fw 5′-CTGGACAATTCCAGCCATCT-3′ and Rev 5′-GGGTGGCAACTCTGACAAAT-3′. 18S RNA was simultaneously amplified using the primers: Fw 5′-AAACGGCTACCACATCCAAG-3′ and Rev 5′-CCTCCAATGGATCCTCGTTA-3′.

The real-time RT-PCR was performed using the double-stranded DNA binding dye SYBR Green PCR Master Mix (Fermentas-Thermo Scientific) on an ABI GeneAmp 7000 Sequence Detection System machine, as described by the manufacturer. The instrument, for each gene tested, obtained graphical cycle threshold values automatically. Triplicate reactions were performed for each marker and the melting curves were constructed using Dissociation Curves Software (Applied Biosystems, CA, USA), to ensure that only a single product was amplified.

The primers sequences were: 18S, Fw 5′-CCCACTCGGCACCTTACG-3′ and Rev

5′-TTTCAGCCTTGCGACCATACT-3′; *KDM6B*, Fw 5′-CCTCGAAATCCCATCACAGT-3′ and Rev 5′-GTGCCTGTCAGATCCCAGTT-3′; *EZH2*, Fw 5′-GCCAGACTGGGAAGAAATCTG-3′ and Rev 5′-TGTGTTGGAAAATCCAAGTCA-3′.

### Statistical analysis

Statistical evaluation of the differential analysis was performed by one-way ANOVA and Student’s *t*-test.

## Electronic supplementary material

Supplemental Figures
