# Supplementary material for: Targeting estrogen receptor beta (ERβ) for treatment of ovarian cancer: importance of KDM6B and SIRT1 for ERβ expression and functionality
Source: Oncogenesis. 2018 Feb 9;7(2):15. doi: 10.1038/s41389-018-0027-9 (PMC5833712; doi:10.1038/s41389-018-0027-9)
Supplement: Supplementary file 1 — Supplemental Figures [file 41389_2018_27_MOESM1_ESM.pdf]

Figure S1. Additive effect of KB9520 on cisplatin sensitivity in SKOV3 and A2780cis/ERα cells

Percentage of growth inhibition in **A)** SKOV3 and **B)** A2780cis**/**ERα cells after 24 hours treatment with different doses of cisplatin, in the range of 1 μM to 160 μM alone or in combination with 10 nM of KB9520 or PPT. Results are expressed as mean ± s.d.) of three independent experiments. *p ≤ 0.05.

Figure S2. Additive effect of KB9520 on paclitaxel sensitivity in A2780cis cells

Percentage of growth inhibition in A2780cis cells after 24 hours treatment with different doses of paclitaxel, in the range of 10 nM to 1 μM alone or in combination with 10 nM of KB9520 or PPT. Results are expressed as mean ± s.d. of three independent experiments. *p ≤ 0.05.

Figure S3. KB9520 treatment induces KDM6B expression in A2780cis cells

Real time-PCR analyses of *KDM6B* and *EZH2* expression in A2780cis cells treated or not with 10nM KB9520 for 2, 4 or 6 hours. 18S rRNA was used as housekeeping gene. Results are expressed as mean ± s.d. of three independent experiments. *p ≤ 0.05.

Figure S4. Role of KDM6B for A2780cis cell response to cisplatin in combination with KB9520

Cell cycle analysis of A2780cis cells transfected with **A**-**C)** non specific or **D)** *KDM6B* specific siRNAs and treated 24 hours with 40 μM of cisplatin alone or in combination with 10 nM of KB9520. After treatments, cells were stained with propidium iodide and analyzed for cellular DNA content by flow cytometry. Exemplificative histograms that plot cell count versus DNA content are reported for each treatment. The percentages of cells in sub-G1 are reported below each plot.

Figure S5. KB9520 treatment does not induce ERα acetylation

Immunoprecipitation of ERα, from lysates of A2780cis cells treated or not for 2 hours with 10nM of KB9520; lysine acetylation and co-immunoprecipitated proteins were detected by Western blot analyses using the respective antibodies (Ac Lys, ERα and p300).

Figure S6. Role of SIRT1 for ERβ de-acetylation in A2780cis cells

A) Immunoprecipitation of ERβ, from lysates of A2780cis cells treated or not for 2 or 4 hours with 10nM of KB9520 alone or in combination with 10μM EX527; lysine acetylation and immunoprecipitated ERβ were detected by Western blot analyses using the respective antibodies. **B)** Immunoprecipitation of p300, from lysates of A2780cis cells treated or not for 2 hours with 10nM of KB9520 alone or in combination with 10μM EX527; lysine acetylation and co-immunoprecipitated proteins were detected by Western blot analyses using the respective antibodies (p300, SIRT1 and ERβ).

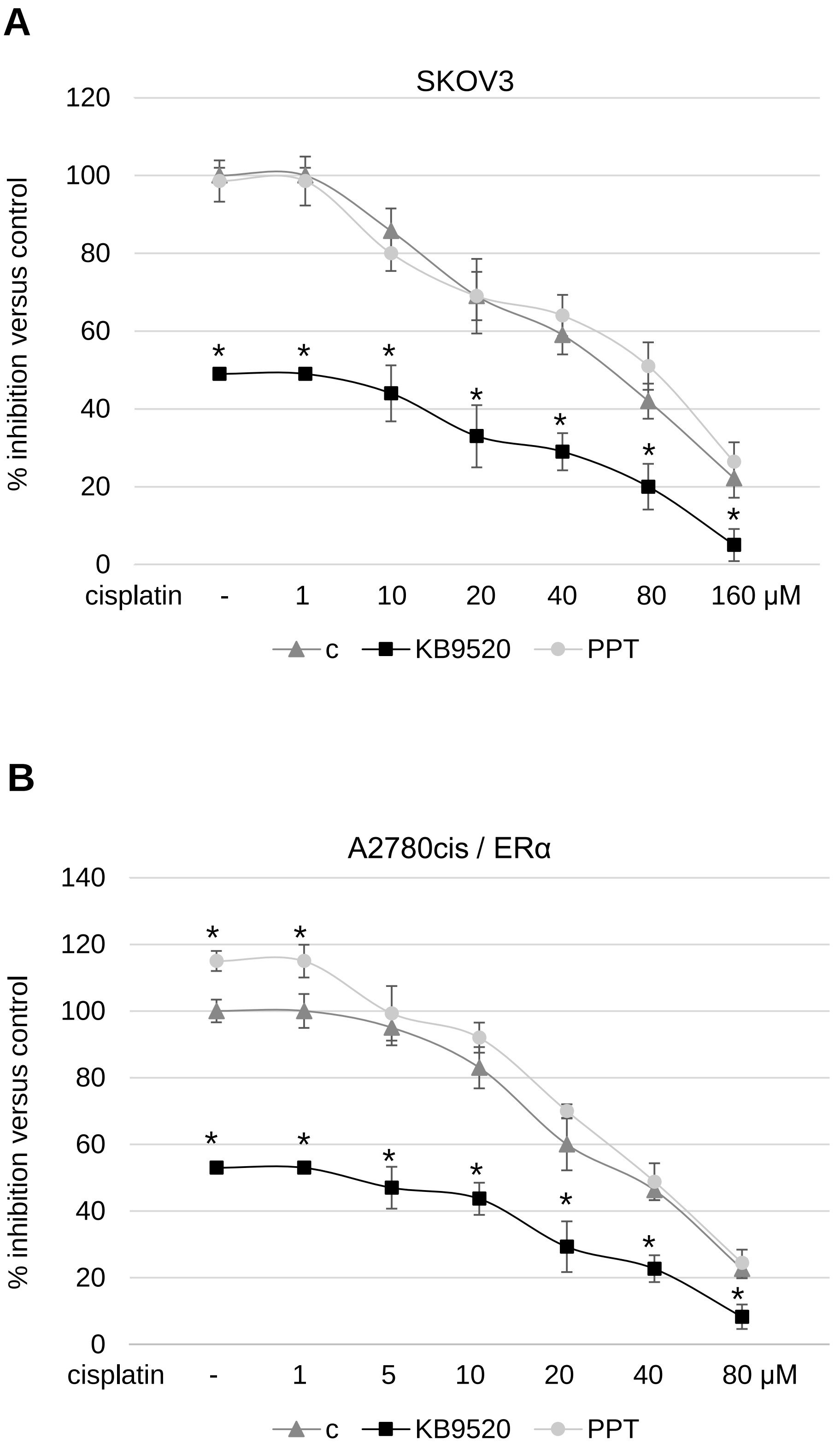
A
SKOV3
120
100
80
60
40
20
0
% inhibition versus control
cisplatin - 1 10 20 40 80 160 µM
c
KB9520
PPT
B
A2780cis / ERα
140
120
100
80
60
40
20
0
% inhibition versus control
cisplatin - 1 5 10 20 40 80 µM
c
KB9520
PPT

Figure S1

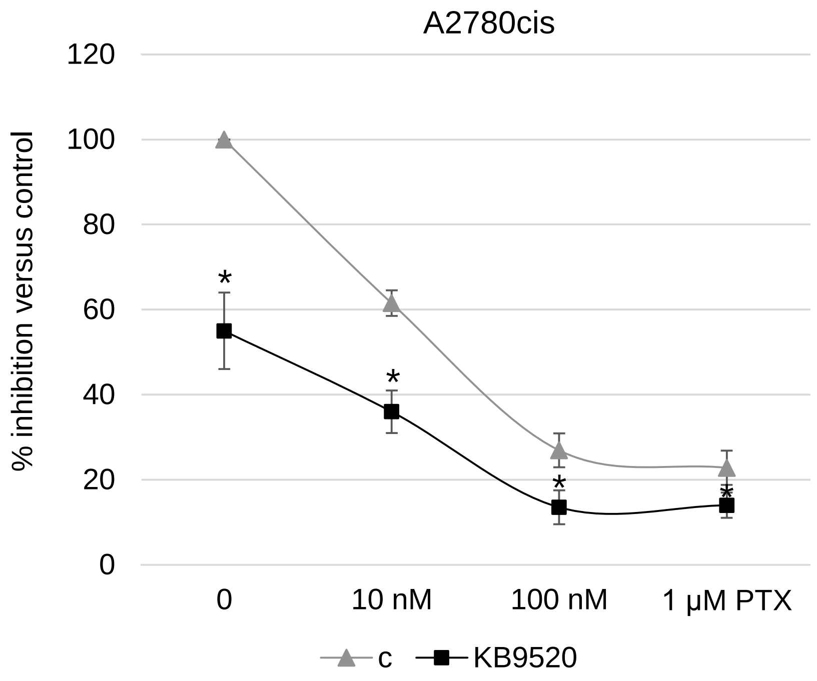

A2780cis
% inhibition versus control
120
100
80
60
40
20
0
*
*
*
*
0
10 nM
100 nM
1 µM PTX
c
KB9520

Figure S2

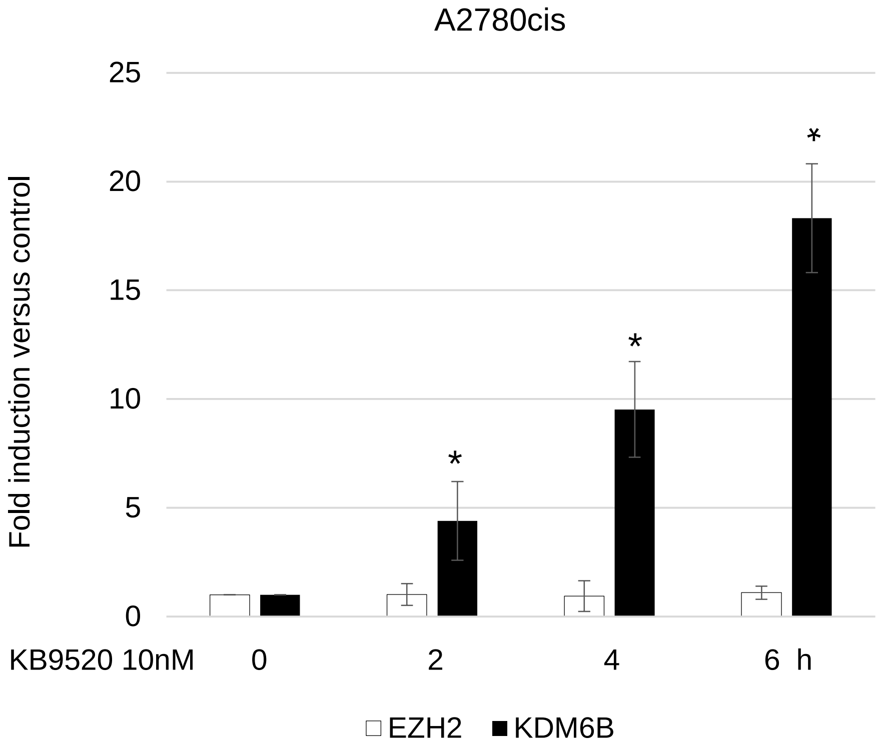
A2780cis
Fold induction versus control
25
20
15
10
5
0
*
*
*
KB9520 10nM
0
2
4
6 h
EZH2
KDM6B

Figure S3

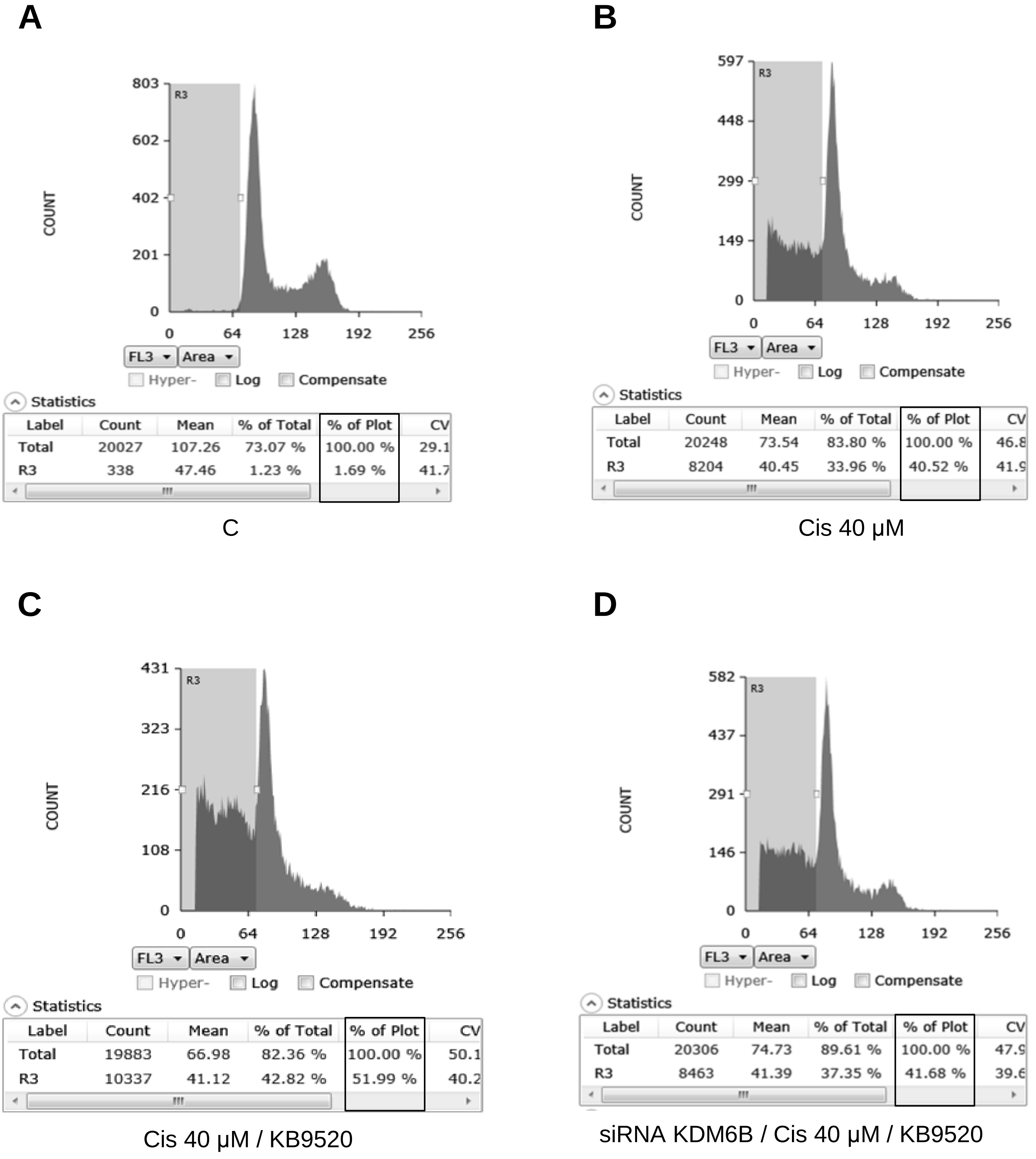
A
R3
COUNT
803
602
402
201
0
0 64 128 192 256
FL3 Area
Hyper- Log Compensate
Statistics
Label Count Mean % of Total % of Plot CV
Total 20027 107.26 73.07 % 100.00 % 29.1
R3 338 47.46 1.23 % 1.69 % 41.7
C
B
R3
COUNT
597
448
299
149
0
0 64 128 192 256
FL3 Area
Hyper- Log Compensate
Statistics
Label Count Mean % of Total % of Plot CV
Total 20248 73.54 83.80 % 100.00 % 46.8
R3 8204 40.45 33.96 % 40.52 % 41.9
Cis 40 µM
C
R3
COUNT
431
323
216
108
0
0 64 128 192 256
FL3 Area
Hyper- Log Compensate
Statistics
Label Count Mean % of Total % of Plot CV
Total 19883 66.98 82.36 % 100.00 % 50.1
R3 10337 41.12 42.82 % 51.99 % 40.2
Cis 40 µM / KB9520
D
R3
COUNT
582
437
291
146
0
0 64 128 192 256
FL3 Area
Hyper- Log Compensate
Statistics
Label Count Mean % of Total % of Plot CV
Total 20306 74.73 89.61 % 100.00 % 47.9
R3 8463 41.39 37.35 % 41.68 % 39.6
siRNA KDM6B / Cis 40 µM / KB9520

Figure S4

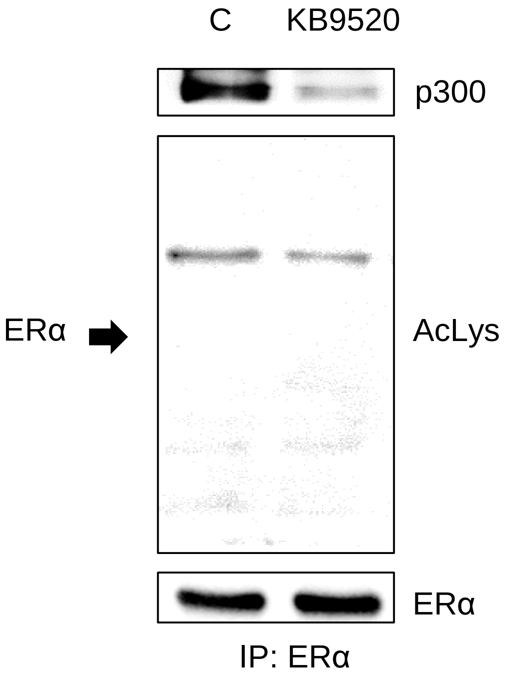

Figure S5

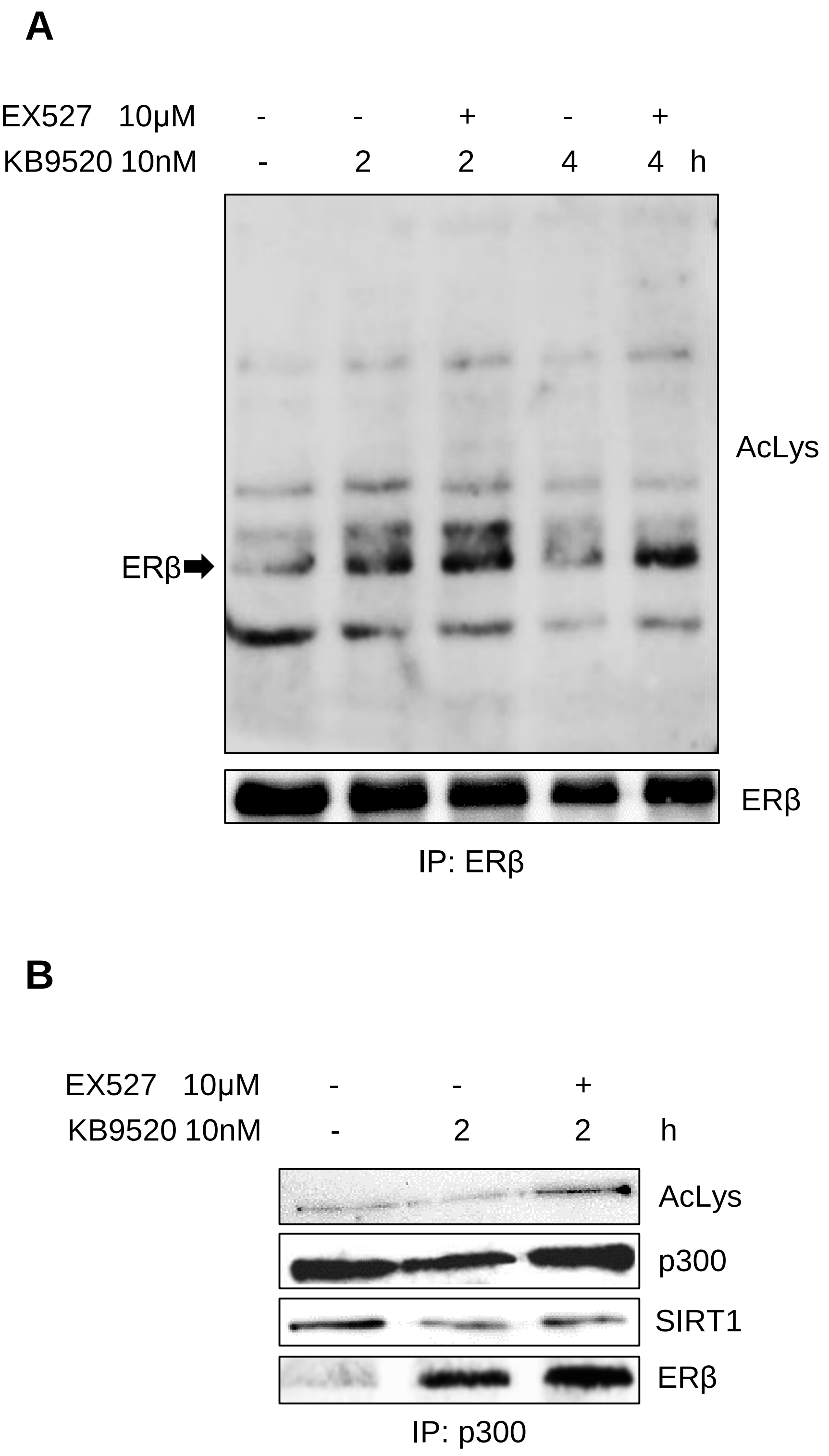
A
EX527 10μM - - + - +
KB9520 10nM - 2 2 4 4 h
AcLys
ERβ
ERβ
IP: ERβ
B
EX527 10μM - - +
KB9520 10nM - 2 2 h
AcLys
p300
SIRT1
ERβ
IP: p300

Figure S6
